# Modeling of metastable phase formation diagrams for sputtered thin films

**DOI:** 10.1080/14686996.2016.1167572

**Published:** 2016-05-04

**Authors:** Keke Chang, Denis Music, Moritz to Baben, Dennis Lange, Hamid Bolvardi, Jochen M. Schneider

**Affiliations:** ^a^ Materials Chemistry, RWTH Aachen University, D-52074Aachen, Germany

**Keywords:** Combinatorial magnetron sputtering, metastable phase formation diagram, thin film growth, surface diffusion distance, Cu–W and Cu–V, 40 Optical, magnetic and electronic device materials, 306 Thin film/Coatings, 300 Processing/Synthesis and Recycling, 401 1st principle calculations, 400 Modeling/Simulations, 403 CALPHAD/Phase field methods, 400 Modeling/Simulations, 307 Kinetics and energy/mass transport, 300 Processing/Synthesis and Recycling

## Abstract

A method to model the metastable phase formation in the Cu–W system based on the critical surface diffusion distance has been developed. The driver for the formation of a second phase is the critical diffusion distance which is dependent on the solubility of W in Cu and on the solubility of Cu in W. Based on comparative theoretical and experimental data, we can describe the relationship between the solubilities and the critical diffusion distances in order to model the metastable phase formation. Metastable phase formation diagrams for Cu–W and Cu–V thin films are predicted and validated by combinatorial magnetron sputtering experiments. The correlative experimental and theoretical research strategy adopted here enables us to efficiently describe the relationship between the solubilities and the critical diffusion distances in order to model the metastable phase formation during magnetron sputtering.

## Graphical abstract

**Figure:**
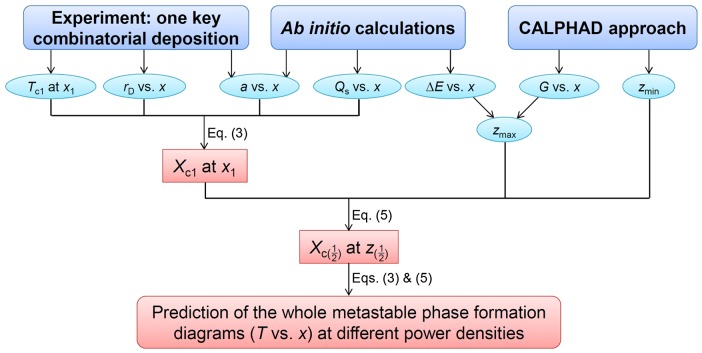

## 1. Introduction

During energetic vapor phase condensation, for example by magnetron sputtering and cathodic arc deposition, the formation of metastable phases is often observed. Generally, this is attributed to kinetically limited growth processes that govern thin film structure evolution.[1–3] For thin films deposited at low temperatures, surface diffusion is the underlying physical mechanism that controls metastable phase formation.[1–3] The atomic mobility can be studied using the temporal dependence of the surface diffusion distance of an atom as given by Einstein [4]:

(1) $$X=\sqrt{2D_{\text{s}}t}$$

where *X* is the diffusion distance, *D*_s_ is surface diffusivity and *t* is the time. Based on Equation (1), Cantor and Cahn [5] proposed the following equation to describe the deposition rate and temperature dependence of the diffusion distance during thin film deposition:

(2) $$X=\sqrt{2\nu \frac{a}{r_{\text{D}}}}\cdot a\cdot \exp\left(-\frac{Q_{\text{s}}}{2kT}\right)$$

where *ν* is the vibrational frequency of surface atoms (~10^13^ s^−1^ [6]), *a* is the individual jump distance, *r*_D_ is the deposition rate, *Q*_s_ is the activation energy for surface diffusion, *k* is the Boltzmann constant and *T* is the substrate temperature during deposition. Saunders and Miodownik [7] also considered the effect of bulk diffusion and modified Equation (2) accordingly in order to investigate the metastable phase formation of Cu_0.57_Ag_0.43_, Pd_0.58_Rh_0.42_, Cu_0.885_Sn_0.115_ and Cu_0.895_Sn_0.105_ thin films. The surface diffusion distances were calculated as a function of temperature.[7] Guided by the experimental phase formation data, the critical surface diffusion distances were estimated around an experimentally determined phase boundary.[7] Therefore, Saunders and Miodownik [7] have described the metastable phase formation for thin films at experimentally determined phase boundaries. However, no modeling attempt has been undertaken to predict metastable phase formation diagrams covering the whole composition range.

**Figure 1.**
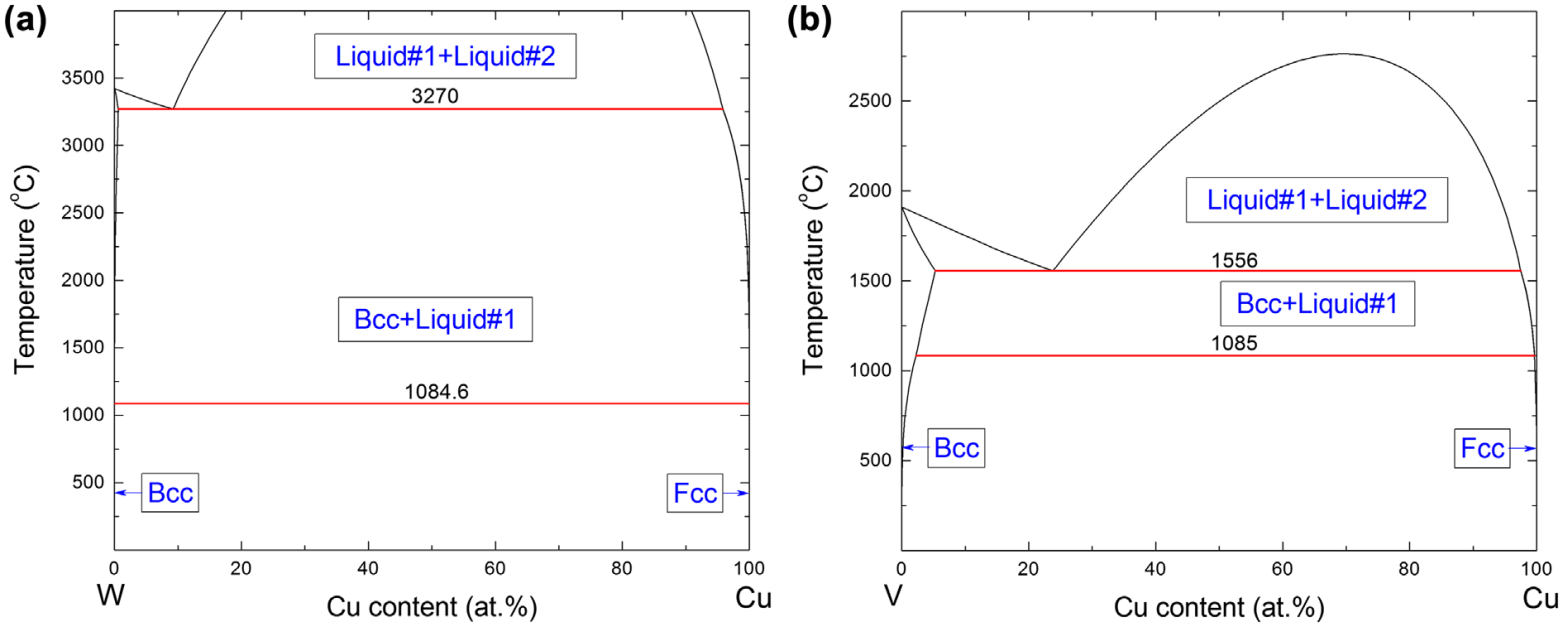
Stable Cu–W (a) and Cu–V [36] (b) phase diagrams calculated using the CALPHAD approach. The Gibbs energy expressions of all the phases are provided in Appendix A.

Metastable phase formation diagrams can be compiled based on experimental phase formation data of individual deposition experiments [8] or from combinatorial thin film synthesis experiments.[9–11] The CALPHAD (Computer Coupling of Phase Diagrams and Thermochemistry) approach provides thermodynamic input to study metastable phase formation [7] and has been applied to describe the metastable solubility limit in a system during thin film deposition, based on Gibbs energy versus composition (G vs. *x*) diagrams.[7,13–15] Using *ab initio* calculations, the metastable solubility limit and formation energy versus composition (E_f_ vs. *x*) can be estimated.[16–18] Together with experimental phase formation data, *ab initio* data has recently been employed to estimate the activation energy for surface diffusion during metastable phase formation.[19] It was demonstrated for sputtered Cu–W thin films that the activation energy data obtained [19] can be utilized to calculate the surface diffusion distance using Equation (2). Recently, attention has been given to immiscible Cu–W and Cu–V systems (for which phase diagrams are shown in Figure 1). The Cu–W thin films investigated by Vüllers and Spolenak [20] show a desirable property combination: W’s high hardness and thermo-mechanical stability and Cu’s excellent thermal conductivity and electrical performance, thereby allowing for possible applications including thermal management, high power and high voltage appliances. The Cu–V thin films feature excellent thermal stability, and have been applied as seed layers for Cu interconnects.[21–23]

The aims of the present work are (1) to develop a model with which to describe the relationship between solubilities and critical diffusion distances based on experimental phase formation data,[19] (2) to predict metastable Cu–W and Cu–V phase formation diagrams and (3) to validate these predictions using additional thin film growth and characterization experiments.

## 2. Experimental and theoretical methods

Cu–V thin films were synthesized by DC magnetron sputtering in an industrial CemeCon 800/9 system (CemeCon AG, Würselen, Germany), where the combinatorial approach [9–12] with split Cu and V targets was applied. The base pressure was less than 10^−4^ Pa and the argon partial pressure was 0.35 Pa during deposition. The distance between target and substrate was 9.5 cm. Si (100) and Al_2_O_3_ (0001) wafers were used as substrates. One set of thin films was deposited at a power density of 0.91 W·cm^−2^ and temperature of 240 °C, respectively. The power density is equal to the power applied to the target, divided by the target size (50 × 8.8 cm^2^). The substrate temperature was measured using three thermocouples clamped to the substrate surface. More information about deposition setup can be found elsewhere.[19] Composition of the samples was analyzed with a scanning electron microscope (SEM; JEOL JSM 6480, JEOL Ltd., Tokyo, Japan) equipped with an EDAX2000 energy dispersive X-ray spectrometer (EDX, EDAX Inc., Mahwah, NJ, USA). The thin film thickness was measured by cross-sectional SEM and the deposition rate was calculated by dividing film thickness by deposition time. Structural analysis was carried out by means of X-ray diffraction (XRD) with a Bruker AXS D8 Discover General Area Diffraction Detector System (GADDS, Bruker AXS GmbH, Karlsruhe, Germany). The strain-free lattice parameters were determined using the sin^2^ *ψ* method.[24,25] These experimental data were then applied as the input in predicting metastable phase formation diagrams for the Cu–V system. Additional combinatorial sputtering processes were carried out at different power densities (0.91, 3.64 and 7.28 W·cm^−2^) and different substrate temperatures (80–340 °C). This phase formation data was used to validate the predictions.

Lattice parameters and formation energetics of body centered cubic (bcc) and face centered cubic (fcc) Cu–V solid solutions were investigated using *ab initio* calculations utilizing the coherent potential approximation (CPA) and special quasi random structure (SQS) approaches. For CPA, the exact muffin tin orbitals (EMTO) formalism [26,27] based on Green’s function [28] and full charge density [29] techniques was used. The generalized gradient approximation (GGA) [30] was applied. The core states were fixed and the total energy was converged within 10^−7^ Ry. For SQS, 16 atoms per unit cell of Cu_*x*_W_1-*x*_ (*x* = 0.0625, 0.125, 0.1875, 0.25, 0.5, 0.75, 0.8125, 0.875 and 0.9375) for both bcc and fcc phases were used for calculations in the Vienna *ab initio* simulation package (VASP).[31] The valence electrons were explicitly treated by projector augmented plane-wave (PAW) potentials.[32] The GGA method was performed using Blöchl corrections for the total energy [33] with a plane-wave cutoff energy of 500 eV and a convergence criterion for the total energy of 0.01 meV. Integration in the Brillouin zone was done on appropriate *k*-points, which was determined after Monkhorst-Pack.[34] Migrations of Cu and V atoms in bcc (110) and fcc (111) surfaces were calculated using *ab initio* calculations in order to obtain surface diffusion activation barriers. A detailed description of the method can be found elsewhere.[19]

The CALPHAD approach was utilized to calculate stable phase diagrams of the Cu–W and Cu–V systems considering the phase equilibria and thermodynamic data from [35] and [36], respectively. FactSage software [37] was utilized for the calculations. Gibbs energy expressions for all of the phases are provided in Appendix A. While differing from [35], generally accepted Gibbs energy functions for pure Cu and W [38] were used in the present calculation. Therefore, the temperature of the invariant reaction (Liquid#2 = bcc + Liquid#1) in the Cu–W phase diagram is 3270 °C, compared to 3240 °C in [35]. As no experimental information is available for this reaction, the calculations in this work, as well as in [35], should therefore be regarded as hypothetical. Moreover, the Gibbs energies for the Cu–V system are identical to those employed in [36].

## 3. Results and discussion

### 3.1. A model to describe the relationship between solubilities and critical diffusion distance

**Figure 2.**
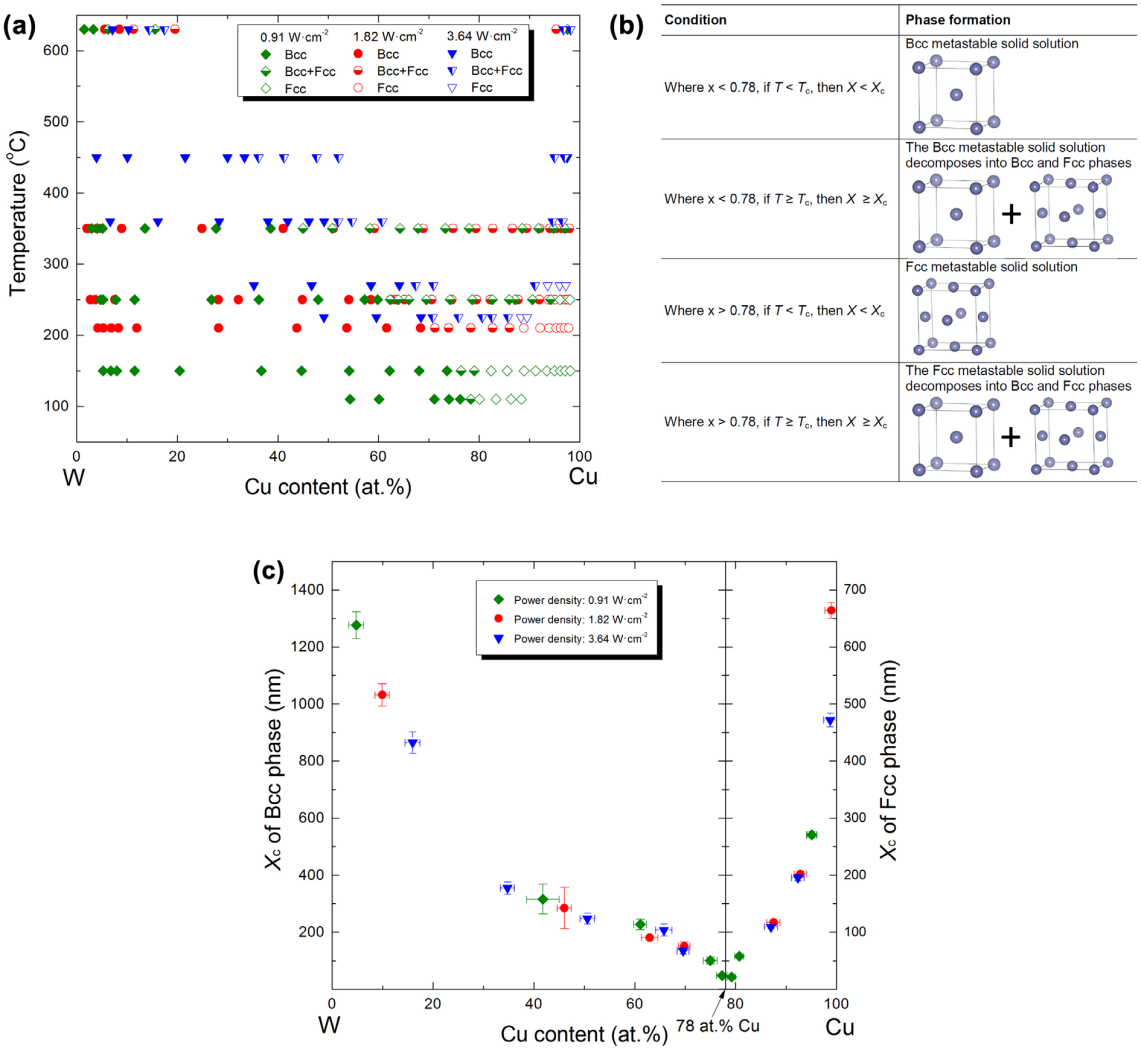
Metastable phase formation in Cu–W thin films: (a) experimental data of thin films grown at different power densities [19]; (b) structure evolution for Cu_*x*_W_1-*x*_ thin films at a certain deposition rate (*T*_c_: critical temperature; *X*_c_: critical surface diffusion distance); and (c) composition dependence of *X*_c_ at the bcc and fcc surfaces.

Experimental phase formation data in the Cu–W thin films were obtained,[19] as shown in Figure 2(a). The maximum solid solubility limit of Cu in bcc-W is found to be approx. 78 at.%. It was suggested that as *X* reaches a critical diffusion distance (*X*_c_), the formation of the second phase and hence decomposition is observed experimentally.[19] Equation (2) can thus be rewritten including *X*_c_:

(3) $$X_{\text{c}}=\sqrt{2\nu \frac{a}{r_{\text{D}n}}}\cdot a\cdot \exp\left(-\frac{Q_{\text{s}}}{2kT_{\text{c}}}\right)$$

where *T*_c_ denotes the critical temperature for each Cu_*x*_W_1-*x*_ composition at a certain deposition rate (*r*_D*n*_). The phase formation data (Figure 2(a)) are subsequently summarized in Figure 2(b). If the substrate temperature is less than *T*_c_, the diffusion distance of atoms on bcc or fcc surfaces is smaller than *X*_c_, meaning that the formation of thermodynamically stable phases is prevented by kinetic limitations in that the atomic mobility is insufficient. If the temperature increases to a value equal to or larger than *T*_c_, atomic mobility is enhanced, causing decomposition of the metastable solid solutions into bcc + fcc phases. Thus, being able to quantify the critical diffusion distance is essential for modeling metastable phase formation diagrams. Using Equation (3), the composition dependence of *X*_c_ was obtained, as is shown in Figure 2(c). Activation energies (*Q*_s_) derived from experimental data [19] were used in the calculations. It is interesting to note that all experiments result in a unified *X*_c_ vs composition behavior, suggesting that *X*_c_ is in fact a temperature-independent and deposition rate-independent property.

**Figure 3.**
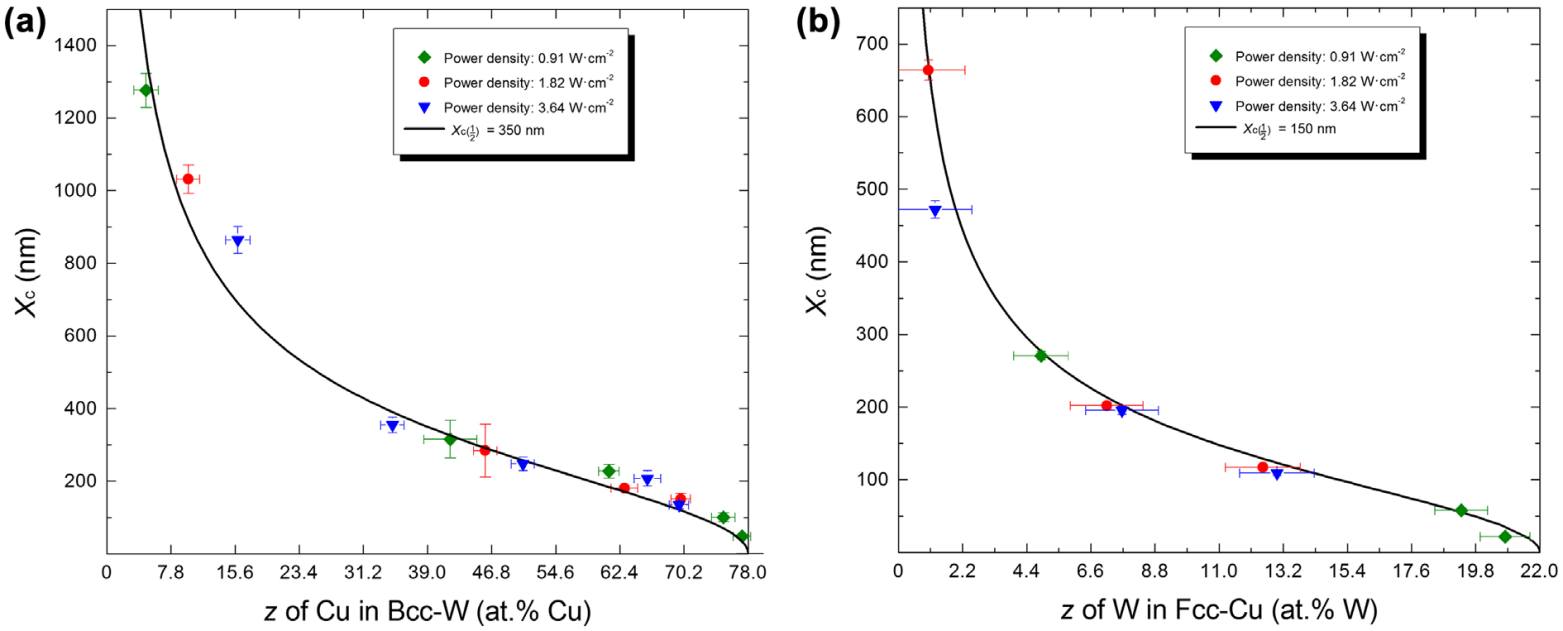
*X*_*c*_ vs. *z* plot: experimental data and fitted curves using Equation (5) for both bcc (a) and fcc (b) phases in the Cu–W system.

In order to model metastable phase formation (Figure 2(a)), it is essential to determine the composition dependence of *X*_c_. In other words, if *X*_c_ for each *x* in Cu_*x*_W_1-*x*_ is known, one can obtain the corresponding *T*_c_ using Equation (3) and the whole metastable phase formation diagram can be calculated. The mutual stable solid solubility at the growth temperature between Cu and W is negligible [35], as shown in the calculated Cu–W phase diagram (Figure 1(a)). However, metastable solid solubility (*z*) of Cu in bcc-W and W in fcc-Cu is obtained in magnetron sputtering experiments [19]. *z* is the composition of the boundary between single-phase and two-phase regions. The maximum metastable solubilities (*z*_max_) for Cu in bcc-W and W in fcc-Cu at 110 °C and the power density of 0.92 W·cm^−2^ are about 78.0 at.% Cu and 22.0 at.% W [19], respectively. To compile a metastable phase formation model, the relationship of *X*_c_ and *z* needs to be determined, based on experimental data. The *X*_c_ vs. *x* diagram (Figure 2(c)) has been transformed into two diagrams (Figure 3(a) and (b)) describing *X*_c_ vs. *z* for the bcc and fcc phases, respectively. Each dataset shows a similar trend. Independent of crystal structure, the relationship of *X*_c_ and *z* can be fitted well using the following sigmoid function:

(4a) $$z=z_{\min}+\frac{z_{\max}-z_{\min}}{1+{\left(\frac{X_{c}}{A}\right)}^{B}},$$

(4b) $$i.e.\;X_{c}=A\cdot \sqrt[{B}]{\frac{z_{\max}-z}{z-z_{\min}}},$$

where *z*_max_ denotes maximum metastable solid solubility and *z*_min_ denotes the stable solid solubility from the equilibrium phase diagrams (Figure 1(a) and (b)). *A* is the critical diffusion distance at half metastable solid solubility (when $z=\frac{1}{2}\left(z_{\text{max}}+z_{\text{min}}\right),X_{c}=A$) and will be denoted as $X_{c(\frac{1}{2})}$. In the Cu–W system, *z*_min_ ≈ 0 for both bcc and fcc phases (see Figure 1(a)). According to experimental data [19], *z*_max_ ≈ 78 at.% Cu for bcc phase and *z*_max_ ≈ 22 at.% W for fcc phase. By fitting each dataset in Figure 3(a) and (b), the value of *B* is obtained ≈2 for both bcc and fcc phases and is henceforth assumed to be a constant. Different values of $X_{c(\frac{1}{2})}$ for the fitted curves can be obtained, and are shown in Figure 3. Therefore, Equation (4b) can be written in the following form:

(5) $$X_{c}=X_{c(\frac{1}{2})}\cdot \sqrt{\frac{z_{\max}-z}{z-z_{\min}}}$$

This model defines the surface diffusion required for atoms to drive second phase formation and reveals the relationship between solubilities and the critical diffusion distance. If *z* = *z*_max_, meaning the phase is highly unstable, then *X*_*c*_ = 0, indicating that the atoms need only a minute diffusion distance in order to form a second phase. If *z* = *z*_min_, then *X*_*c*_ = +∞, denoting that the phase is thermodynamically stable and that the system is always in its equilibrium state.

### 3.2. A model based research strategy for predicting metastable phase formation diagrams

**Figure 4.**
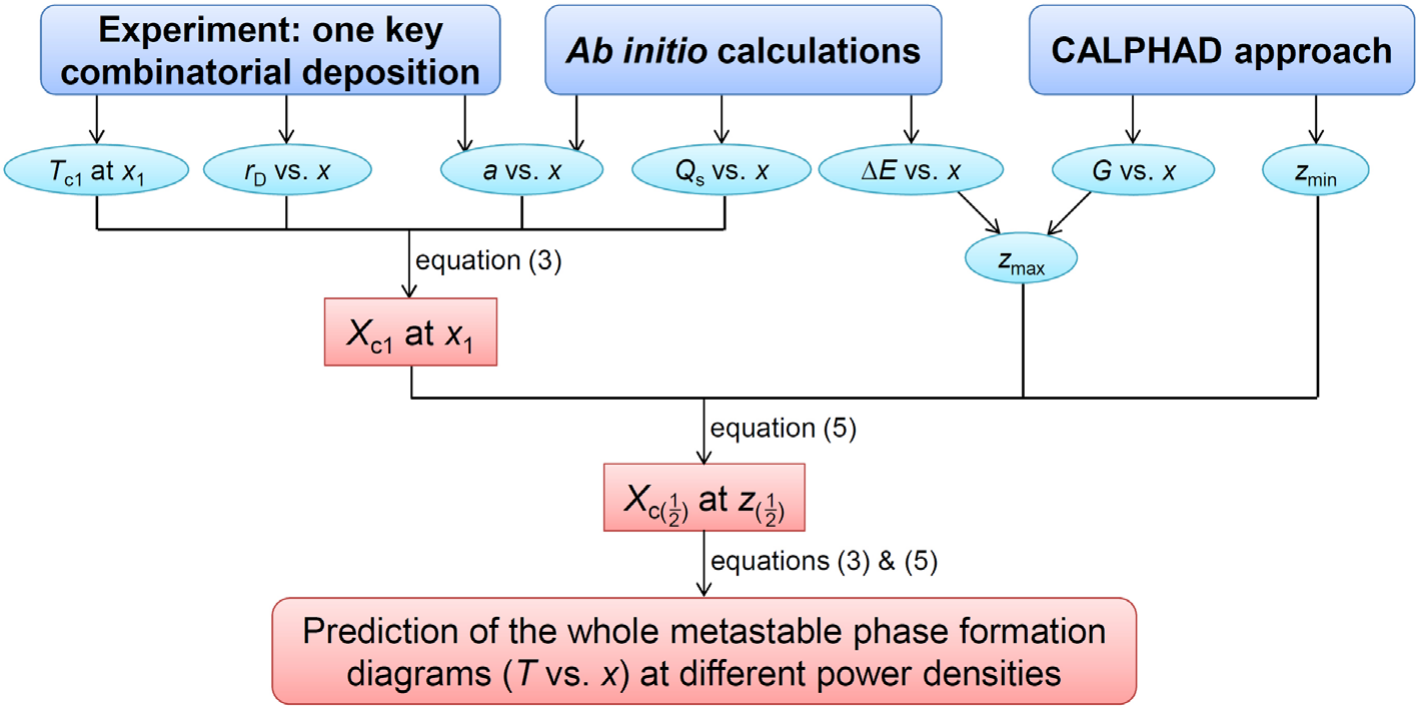
Flowchart of the present research strategy to predict metastable phase formation diagrams for sputtered thin films.

A combination of Equations (3) and (5) enables the prediction of metastable phase formation diagrams, based on a correlative experimental and theoretical research strategy. This method is delineated in the flowchart in Figure 4. One can calculate the diagram at a certain power density with input of the following variables: *T*_c1,_ *r*_D_, *a*, *Q*_s_, *z*_max_ and *z*_min_. By varying the power density, *r*_D_ is affected and the metastable phase formation diagram changes accordingly. Assuming that *r*_D_ is directly proportional to power density, the diagrams at different power densities can be predicted. In Figure 4, *T*_c1_ denotes one critical temperature determined by experiments. *r*_D_ and *a* can also be acquired using experiments. *Ab initio* calculations can be used to obtain *a*, *Q*_s_ and *z*_max_, while the CALPHAD approach can be applied to describe both *z*_max_ and *z*_min_.

Generally, one key combinatorial deposition together with the above-described calculations is needed to map metastable phase formation diagrams, provided the following necessary requirement is fulfilled: the experimental phase formation data has to cover the complete composition space, containing all of the phase boundaries to be modeled. For instance, one cannot obtain a critical temperature for the fcc phase based only on experimental data at 350 °C, because all of the data on the Cu-rich side exhibits bcc + fcc two phase structures (see Figure 2(a)).

### 3.3. Modeling of the Cu–W system

**Figure 5.**
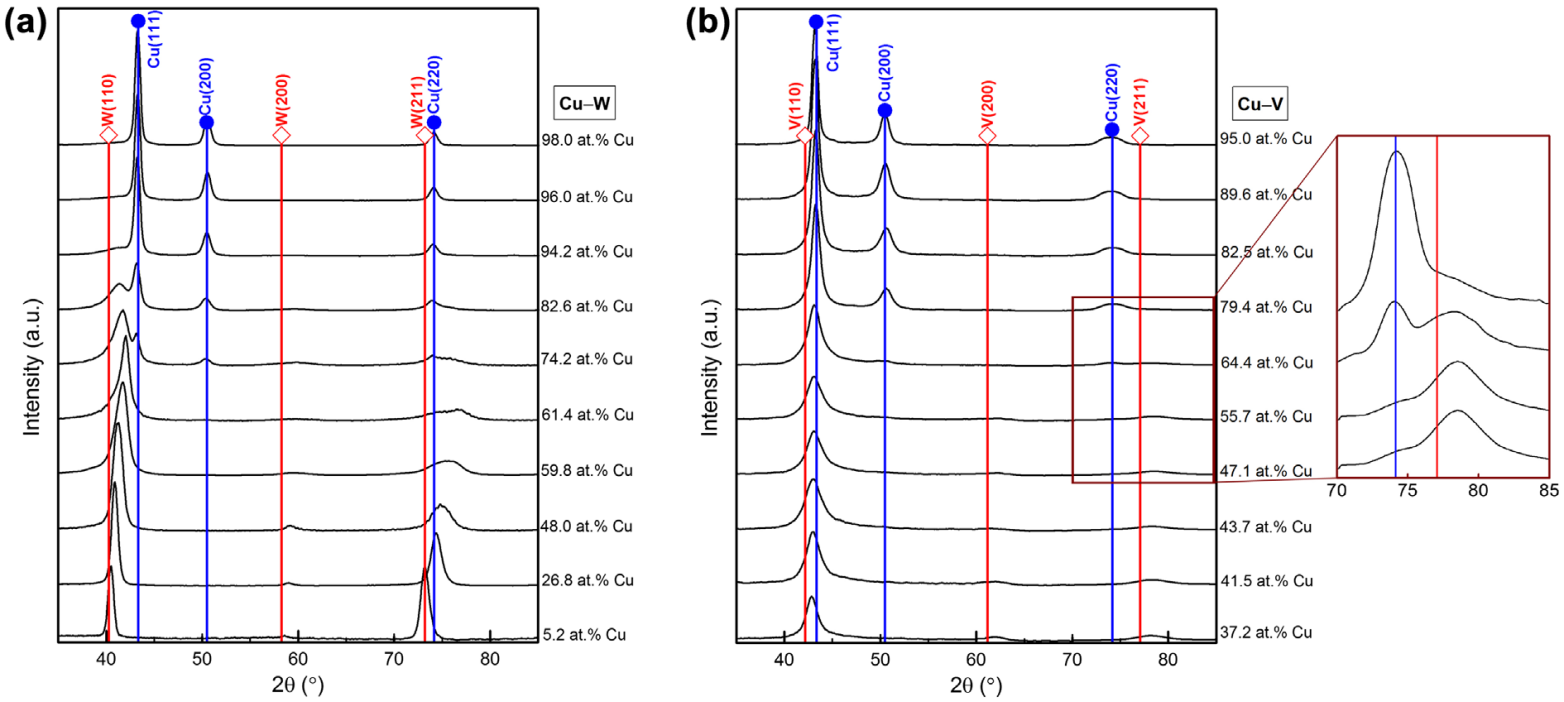
XRD profiles of (a) Cu–W thin films deposited at a temperature of 250 °C and power density of 0.91 W·cm^−2^; (b) Cu–V thin films deposited at a temperature of 240 °C and power density of 0.91 W·cm^−2^. Reference 2*θ* positions for the pure elements are taken from references [39–41].

The phase formation data obtained from one deposition performed at a temperature of 250 °C and power density of 0.91 W·cm^−2^ have been selected to model the Cu–W system. XRD profiles of the Cu–W thin films are shown in Figure 5(a), where 2*θ* positions of pure Cu [39] and W [40] are added as references. Thin films with Cu concentrations below 59.8 at.% are composed of a bcc solid solution, while those with Cu concentrations above 96.0 at.% are composed of an fcc solid solution. The thin films with Cu concentration ranging from 61.4 to 94.2 at.% contain bcc and fcc phases. Therefore, *T*_c1_ is equal to 250 °C at the composition of ~60.6 and ~ 95.1 at.% Cu for the bcc and fcc phases, respectively. Previously,[19] *a*, *z*_max_ and *Q*_s_ have been described by *ab initio* calculations. *z*_min_ equaling zero was obtained here using the CALPHAD approach (Figure 1(a)). It is known that grain size is affected for example by the deposition rate and substrate temperature [42]. The model proposed here is based on the phase formation data only. Microstructural features such as grain size information are not included at present.

**Figure 6.**
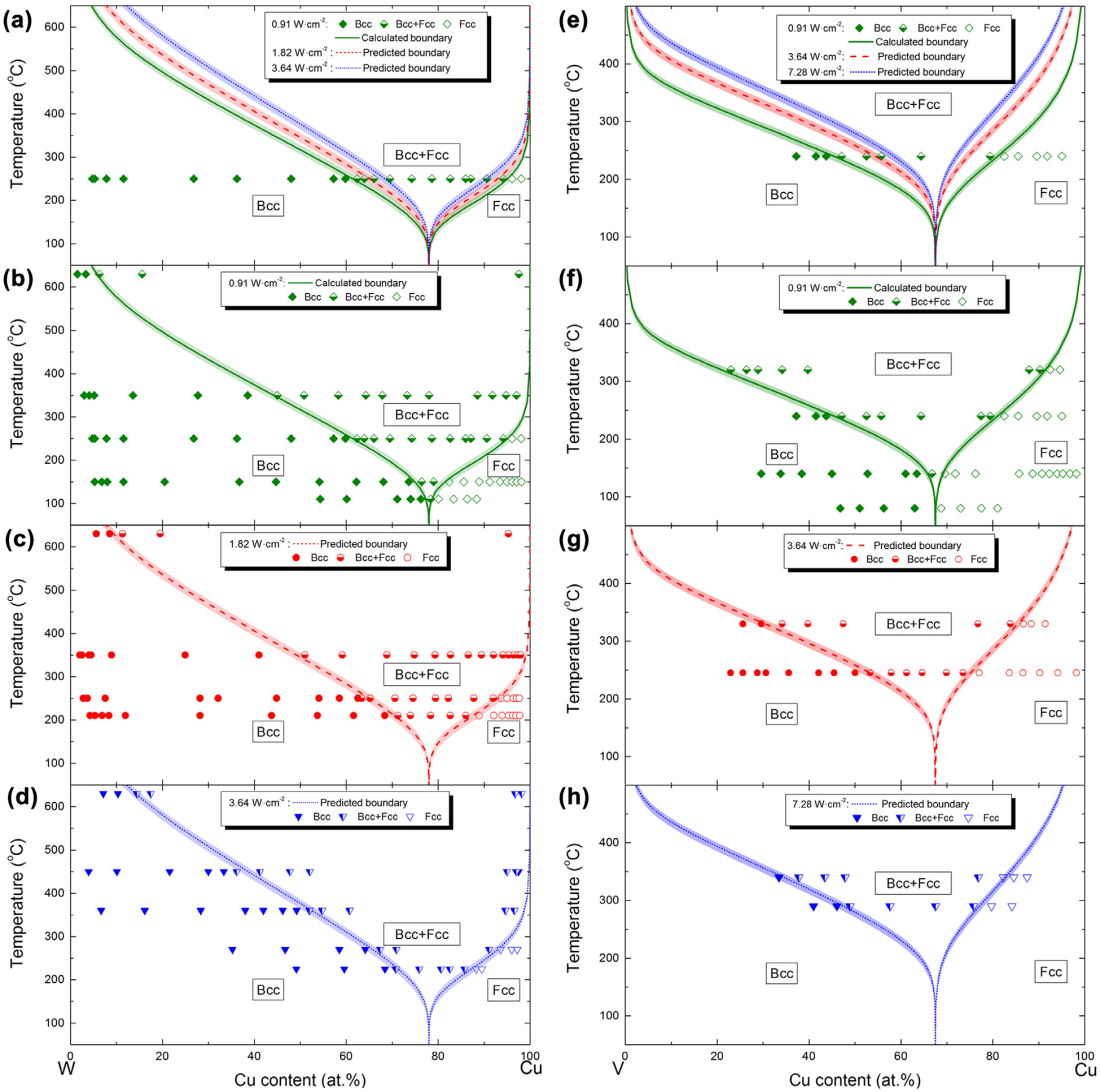
Metastable Cu–W phase formation diagrams: (a) calculated and predicted diagrams using experimental data [19] at a temperature of 250 °C and power density of 0.91 W·cm^−2^; validation using experimental data [19] at power densities of (b) 0.91 W·cm^−2^, (c) 1.82 W·cm^−2^ and (d) 3.64 W·cm^−2^. The shaded phase boundaries indicate temperature fluctuations of ±5°C that occurred during thin film synthesis [19]; metastable Cu–V phase formation diagrams: (e) calculated and predicted diagrams using experimental data at a temperature 240 °C and power density of 0.91 W·cm^−2^; validation using experimental data at power densities of (f) 0.91 W·cm^−2^, (g) 3.64 W·cm^−2^ and (h) 7.28 W·cm^−2^. The shaded phase boundaries indicate temperature fluctuations of ±7 °C that occurred during thin film synthesis.

All the above data was considered, following the strategy outlined in Figure 4. A metastable Cu–W phase formation diagram, based on phase formation data obtained at a power density of 0.91 W·cm^−2^ covering the whole composition range, has been calculated. Based on this model, the position of the phase boundaries has been predicted for power densities of 1.82 and 3.64 W·cm^−2^, as is shown in Figure 6(a). Temperature fluctuations of ± 5 °C that occurred during thin film synthesis [19] are represented in the phase formation diagrams as shaded phase boundaries. Additional experimental data [19] obtained at a power density of 0.91 W·cm^−2^ and substrate temperatures of 110, 150, 350 and 630°C are consistent with the model, as shown in Figure 6(b). The predicted phase formation diagrams for power densities of 1.82 and 3.64 W·cm^−2^ agree very well with the experimental phase formation data [19] depicted in Figure 6(c) and Figure 6(d), respectively. Therefore, the research strategy proposed here for modeling and predicting metastable phase formation has been validated for the Cu–W system. The evaluation criteria are the positions of the predicted metastable phase boundaries compared to the experimental data.[19]

Depending on the synthesis technology utilized and the deposition parameters employed, the formation of bcc [19,20,43–46] and fcc [19,20,43–51] solid solutions, amorphous [44–51] and a tetragonal phase [44], as well as mixtures thereof [19,20,43–51] are reported in the literature. The correlative experimental and theoretical research strategy proposed here for modeling the metastable phase formation during magnetron sputtering is clearly not limited to fcc and bcc solid solutions: Provided that the formation of metastable phases other than fcc and bcc solid solutions, for example amorphous phases, are observed experimentally and utilized as input data for the model, the formation of these metastable phases can be predicted.

### 3.4. Modeling of the Cu–V system

The same research strategy developed for and applied to the Cu–W system was also utilized to model and predict metastable phase formation within the Cu–V system. To that end, combinatorial deposition of Cu_*x*_V_1-*x*_ thin films was performed at a temperature of 240 °C and power density of 0.91 W·cm^−2^. XRD profiles of the Cu–V thin films are shown in Figure 5(b) and 2θ positions of pure Cu [39] and V [41] are added as references. A bcc solid solution is observed for Cu concentrations below 43.7 at.%, while a fcc solid solution forms at Cu concentrations above 82.5 at.%. Thin films with Cu concentrations ranging from 47.1 to 79.4 at.% comprise a mixture of bcc and fcc phases. Therefore, *T*_c1_ is equal to 240 °C at the composition of ~45.4 and ~81.0 at.% Cu for the bcc and fcc phases, respectively. *r*_D_ equaling 8.5·*x* + 0.5 (Å/s) was acquired. As for the Cu–W system using the CALPHAD approach, *z*_min_ is found to be zero (Figure 1(b)). The *ab initio* results of *a*, *z*_max_ and *Q*_s_ for the Cu–V system are shown in Appendix B.

Following the research strategy specially validated for the Cu-W system (see Figure 4), metastable Cu–V phase formation diagrams were calculated, based on experimental phase formation data obtained at a power density of 0.91 W·cm^−2^ and a substrate temperature of 240 °C. This dataset fulfils the necessary requirement outlined above for modeling the metastable phase formation: the experimental data have to cover the complete composition space, thereby containing all phase boundaries to be modeled. On the basis of this model, phase formation diagrams at power densities of 3.64 and 7.28 W·cm^−2^ have been predicted, as shown in Figure 6(e). The shaded phase boundaries indicate temperature fluctuations of ±7 °C that occurred during thin film synthesis. Supplementary combinatorial depositions of Cu_*x*_V_1-*x*_ thin films were carried out at a power density of 0.91 W·cm^−2^ and substrate temperatures of 80, 140 and 320 °C. The prediction based on the growth data obtained at 240 °C agrees very well with the experimental phase formation data obtained at substrate temperatures of 80, 140 and 320 °C, as shown in Figure 6(f). Further phase formation data obtained from depositions at power densities of 3.64 and 7.28 W·cm^−2^ are shown in Figure 6(g) and 6(h), respectively. These data also agree very well with the initial prediction. Hence, the predictive capability of the modeling strategy proposed here has been validated, as has the Cu–W system for the Cu–V system.

## 4. Conclusions

In this work, the following conclusions have been drawn:

(1) A model has been derived to describe the critical diffusion distance on the basis of experimental data for the Cu–W system, which is solubility dependent. It describes the surface diffusion required for atoms to drive the formation of a second phase.
(2) A strategy to predict metastable phase formation diagrams for sputtered thin films based on one single combinatorial magnetron sputtering experiment, CALPHAD and *ab initio* calculations is proposed. The necessary requirement for the prediction is that the phase formation data cover the complete composition space containing all phase boundaries to be modeled.
(3) The predictive capabilities of the research strategy proposed here have been validated for both the Cu–W and Cu–V systems using additional experimental phase formation data, changing deposition temperature by up to 380 °C and deposition power by up to a factor of eight.

The correlative experimental and theoretical research strategy proposed here provides an efficient way to map metastable phase formation diagrams for sputtered thin films and hence expands the capabilities for future design of metastable thin film materials.

## Disclosure statement

No potential conflict of interest was reported by the authors.

## Notes on Contributors

*Keke Chang* is a postdoctoral researcher at Materials Chemistry, RWTH Aachen University.

*Denis Music* is ab initio group leader at Materials Chemistry, RWTH Aachen University.

Moritz *to Baben* was a postdoctoral researcher at RWTH Aachen University. He currently works at GTT-Technologies, Germany.

*Dennis Lange* received his Master Degree at RWTH Aachen University and his Doctoral Degree at Ecole Polytechnique in Palaiseau (Île-de-France). He currently works as a project leader at the Herzogenrath R&D Center of Saint-Gobain, Germany.

*Hamid Bolvardi* received his doctoral degree at RWTH Aachen University. He currently works as a project manager R&D Tools at Oerlikon Surface Solutions AG, Liechtenstein.

*Jochen M. Schneider*, Ph.D., is Professor of Materials Chemistry at RWTH Aachen University, Germany. Formerly at Linköping University, Sweden, and Northwestern University, USA, Jochen received his Ph.D. degree in surface engineering from Hull University, UK. His research interest is the materials science of thin films grown by plasma-assisted vapor deposition. Jochen has been awarded the Sofya Kovalevskaya Prize by the Alexander von Humboldt Foundation and was named Fellow of AVS in 2013 and Max Planck Fellow and RWTH fellow in 2015.

## Figures and Tables

**Figure B1.**
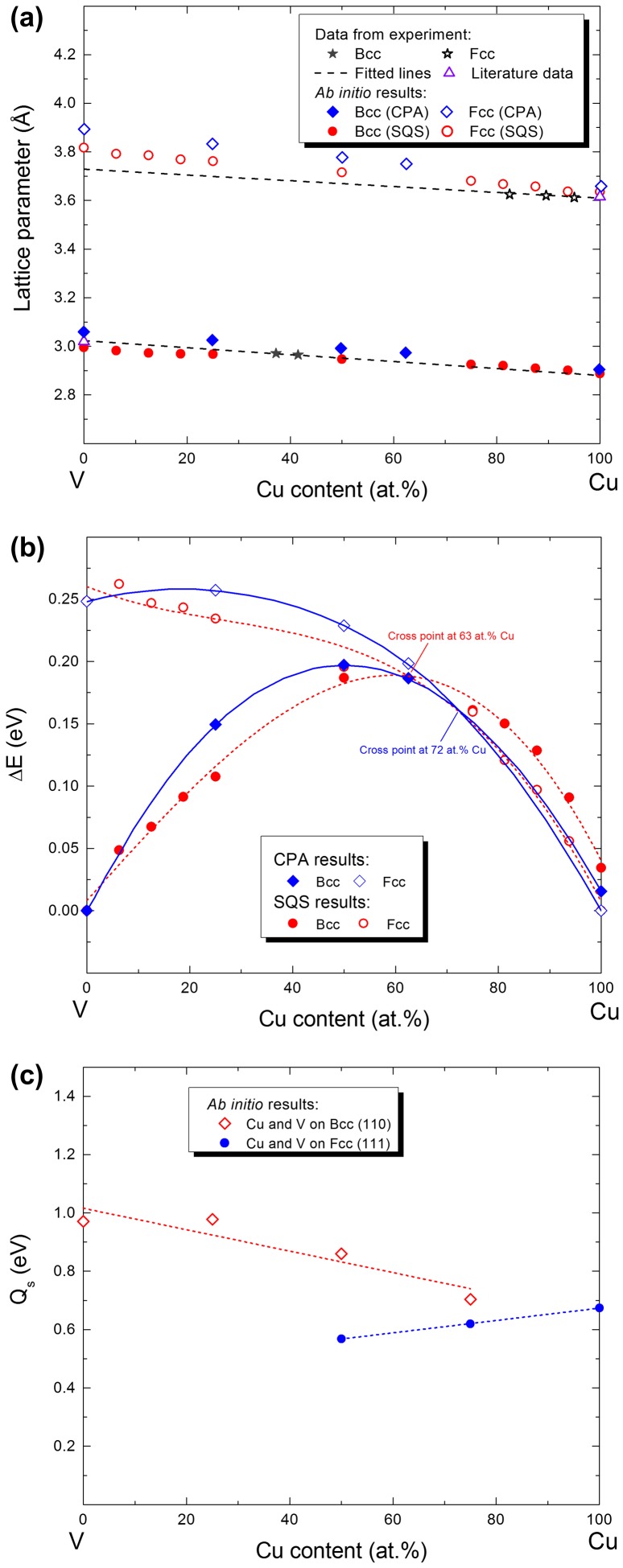
*Ab initio* results of Cu_*x*_V_1-*x*_ as a function of composition: (a) lattice parameters (compared with experimental data), (b) formation energies (Δ*E*) of bcc and fcc phases, (c) activation barriers (*Q*_s_) of Cu and V atoms at bcc (110) and fcc (111) surfaces. Experimental lattice parameters of fcc-Cu and bcc-V are taken from [52,53].

## Appendix A.

Thermodynamic data of the Cu–W and Cu–V systems

The liquid, bcc and fcc phases in the Cu–W and Cu–V systems are described using an ideal solution model.[35,36] Gibbs energy expressions of the Cu–V system are identical to those in [36]. While differing from [35], Gibbs energies for the pure Cu and W are taken from a generally accepted SGTE compilation.[38] Interaction parameters of the liquid Cu_x_W_1-x_ phase [36] are used, while those of bcc and fcc Cu_x_W_1-x_ phases are adjusted on the basis of *ab initio* formation enthalpy data in a previous work.[19] Gibbs energy functions for all of the phases used to calculate the Cu–W and Cu–V phase diagrams (Figure 1) are listed below. Note that quantities are in J mol^–1^ and temperature (*T*) is in K.

(1) Gibbs energies of pure elements:

G(fcc,Cu) =

– 7770.458 + 130.485235·*T* – 24.112392·*T*·ln(*T*) – 0.00265684·*T* ^2^ + 1.29223·10^–7^·*T* ^3^ + 52478·*T* ^–1^ (298.15 K < *T* < 1357.77 K)

– 13542.026 + 183.803828·*T* – 31.38·*T*·ln(*T*) + 3.64167·10^29^·*T* ^–9^ (1357.77 K < *T* < 3200 K)

G(bcc, Cu) =

G(fcc,Cu) + 4017 – 1.255·*T* (298.15 K < *T* < 3200 K)

G(Liquid, Cu) =

G(fcc,Cu) + 12964.736 – 9.511904·*T* – 5.849·10^–21^·*T* ^7^ (298.15 K < *T* < 1357.77 K)

G(fcc,Cu) + 13495.481 – 9.922344·*T* – 3.642·10^29^·*T* ^−9^ (1357.77 K < *T* < 3200 K)

G(bcc,W) =

– 7646.311 + 130.4·*T* – 24.1·*T*·ln(*T*) – 0.001936·*T* ^2^ + 2.07·10^–7^·*T* ^3^ – 5.33·10^–11^·*T* ^4^ + 44500·*T* ^–1^

(298.15 K < *T* < 3695 K)

– 82868.801 + 389.362335·*T* – 54·*T*·ln(*T*) + 1.528621·10^33^·*T* ^–9^ (3695 K < *T* < 6000 K)

G(fcc,W) =

G(bcc,W) + 19300 + 0.63·*T* (298.15 K < *T* < 6000 K)

G(Liquid, W) =

G(bcc,W) + 52160.584 – 14.10999·*T* – 2.713468·10^–24^·*T* ^7^ (298.15 K < *T* < 3695 K)

G(bcc,W) + 52432.75 – 14.187335·*T* – 1.528621·10^33^·*T* ^–9^ (3695 K < *T* < 6000 K)

G(bcc,V) =

– 7930.43 + 133.346053·*T* – 24.134·*T*·ln(*T*) – 0.003098·*T* ^2^ + 1.2175·10^–7^·*T* ^3^ + 69460·*T* ^–1^

(298.15 K < *T* < 790 K)

– 7967.842 + 143.291093·*T* – 25.9·*T*·ln(*T*) + 6.25·10^–5^·*T* ^2^ – 6.8·10^–7^·*T* ^3^

(790 K < *T* < 2183 K)

– 41689.864 + 321.140783·*T* – 47.43·*T*·ln(*T*) + 6.44389·10^31^·*T* ^–9^

(2183 K < *T* < 4000 K)

G(fcc,V) =

G(bcc,V) + 7500 + 1.7·*T* (298.15 K < *T* < 6000 K)

G(Liquid, V) =

G(bcc,V) + 20764.117 – 9.455552·*T* – 5.19136·10^–22^·*T* ^7^ (298.15 K < *T* < 2183 K)

G(bcc,V) + 22072.354 – 10.0848·*T* – 6.44389·10^31^·*T* ^–9^ (2183 K < *T* < 4000 K)

(2) Gibbs energies of the Cu_*x*_W_1-*x*_ phases in the Cu–W system.

G(fcc) =

*x*·G(fcc,Cu) + (1 – *x*)·G(fcc,W) + *R*·*T*·[*x*·ln *x* + (1 – *x*)·ln (1 – *x*)] + *x*·(1 – *x*)·120000

G(bcc) =

*x*·G(bcc,Cu) + (1 – *x*)·G(bcc,W) + *R*·*T*·[*x*·ln *x* + (1 – *x*)·ln (1 – *x*)] + *x*·(1 – *x*)·130000

G(Liquid) =

*x*·G(Liquid,Cu) + (1 – *x*)·G(Liquid,W) + *R*·*T*·[*x*·ln *x* + (1 – *x*)·ln (1 – *x*)]

+ *x*·(1 – *x*)·(101000 – *x*·210000)

(3) Gibbs energies of the Cu_*x*_V_1–*x*_ phases in the Cu–V system:

G(fcc) =

*x*·G(fcc,Cu) + (1 – *x*)·G(fcc,V) + *R*·*T*·[*x*·ln *x* + (1 – *x*)·ln (1 – *x*)] + *x*·(1 – *x*)·53650

G(bcc) =

*x*·G(bcc,Cu) + (1 – *x*)·G(bcc,V) + *R*·*T*·[*x*·ln *x* + (1 – *x*)·ln (1 – *x*)] + *x*·(1 – *x*)·42377.8

G(Liquid) =

*x*·G(Liquid,Cu) + (1 – *x*)·G(Liquid,V) + *R*·*T*·[*x*·ln *x* + (1 – *x*)·ln (1 – *x*)]

+ *x*·(1 – *x*)·(56400 – *x*·37000)

## Appendix B.

*Ab initio* results of bcc and fcc phases in the Cu–V system

*Ab initio* calculated lattice parameters and formation energies of Cu–V phases using the CPA and SQS approaches are shown in Figure B1(a) and B1(b), respectively. In contrast to the experimental data (experimental lattice parameters of fcc-Cu and bcc-V are taken from [52,53]), CPA and SQS calculated lattice parameters exhibit a deviation of +0.84% – +4.42% and −1.08% – +2.37%, respectively, which is an acceptable agreement. Solid solubility limits of Cu in bcc-V calculated using CPA and SQS are 72.0 and 63.0 at.% Cu, respectively. An average value (i.e. 67.5 at.% Cu) is considered as *z*_max_ of Cu in bcc-V for modeling the Cu–V metastable phase formation diagrams. Figure B1(c) shows the *ab initio* obtained *Q*_s_ data, which decreases as the metastable solubility of Cu in bcc-V or V in fcc-Cu increases. This trend is expected and similar to that in the Cu–W system.[19]
